# Current status of projects for developing cancer-related clinical practice guidelines in Japan and recommendations for the future

**DOI:** 10.1007/s10147-018-1340-1

**Published:** 2018-08-24

**Authors:** Masafumi Imamura, Koichi Hirata, Michiaki Unno, Kinj Kamiya, Mitsukazu Gotoh, Hiroyuki Konno, Akiko Shibata, Kenichi Sugihara, Arata Takahashi, Masahiko Nishiyama, Kenichi Hakamada, Tsuguya Fukui, Toshiharu Furukawa, Tsunekazu Mizushima, Masamichi Mizuma, Hiroaki Miyata, Masaki Mori, Ichiro Takemasa, Toru Mizuguchi, Toshiyoshi Fujiwara

**Affiliations:** 1 Department of Surgery, Surgical Oncology and Science, Sapporo Medical University, South 1, West 16, Chuo-ku, Sapporo, 060-8543 Japan; 2 Department of Surgery, Tohoku University Graduate School of Medicine, Sendai, Japan; 3 Second Department of Surgery, Hamamatsu University School of Medicine, Hamamatsu, Japan; 4 Osaka General Medical Center, Osaka, Japan; 5 Hamamatsu University School of Medicine, Hamamatsu, Japan; 6 National Cancer Center, Center for Cancer Control and Information Services, Tokyo, Japan; 7 Tokyo Medical and Dental University, Tokyo, Japan; 8 Department of Health Policy and Management, School of Medicine, Keio University, Tokyo, Japan; 9 Department of Molecular Pharmacology and Oncology, Gunma University Graduate School of Medicine, Maebash, Japan; 10 Department of Gastroenterological Surgery, Hirosaki University Graduate School of Medicine, Hirosaki, Japan; 11 St. Luke’s International University, Tokyo, Japan; 12 Keio University Law School, Tokyo, Japan; 13 Department of Gastroenterological Surgery, Graduate School of Medicine, Osaka University, Osaka, Japan; 14 The University of Tokyo, Healthcare Quality Assessment, Tokyo, Japan; 15 Department of Gastroenterological Surgery, Okayama University Graduate School of Medicine, Dentistry and Pharmaceutical Sciences, Okayama, Japan; 16 JR Sapporo Hospital, North 3, East 1, Chuo-ku, Sapporo, 060-0033 Japan

**Keywords:** Cancers, Clinical practice guidelines, Plan-do-check-act cycle, Japan Society of Clinical Oncology

## Abstract

**Background:**

The current status and adoption of cancer-related clinical practice guidelines in Japan has not been elucidated yet. The purpose of this study was to propose roles and suggestions to develop future cancer-related clinical guidelines.

**Methods:**

A questionnaire consisting of four domains with a total of 17 questions was developed. We distributed the questionnaire to 28 specific academic organizations in Japan which have developed any cancer-related clinical practice guidelines and which were funded by the Ministry of Health, Labor, and Welfare.

**Results:**

Most organizations have investigated nationwide dissemination and adoption of clinical practice guidelines. The rate of adoption in clinical practice was estimated at approximately ≥ 70%. However, organizations with smaller budgets reported surveying approximately 60% of the time, whereas the ones with larger budgets reported approximately 100% success in surveying about their guidelines. The presidents of the organizations agreed that a new organization operated directly by the national government was necessary.

**Conclusion:**

In Japan, to develop cancer-related clinical practice guidelines, a study of clinical validation is necessary. Sufficient funds must be available to support the project to maintain and revise the guidelines. Furthermore, legal and ethical issues should be solved before establishing any registry system.

## Introduction

Many clinical practice guidelines are developed worldwide in various fields including medicine, oncology, and surgery. The essential purpose of creating cancer-related clinical practice guidelines is to improve public knowledge and understanding for improving the quality of cancer treatment [1–3]. To date, we have focused on promoting medical recommendations and clinical evidence in the guidelines. In addition, we have also worked to validate their effectiveness after publishing them in clinical fields. However, most levels of clinical evidence were not high enough to support clinical practice guidelines and their recommendations. Therefore, additional clinical studies are needed to provide more reliable and validated medical evidence.

Cancer-related clinical practice guidelines are becoming popular in the public despite the transition of the medical environment in Japan. In this situation, the guidelines should be objective and sequential for reflecting a high medical consciousness in the public. Therefore, we are finding more situations where we need to establish a system to develop new guidelines while maintaining the existing clinical practice guidelines [4].

Therefore, we attempted to clarify the coordination among the existing organizations that disseminate cancer-related clinical practice guidelines in Japan. We also proposed investigating what types of problems organizations might be encountering under the current circumstances. We developed a questionnaire for academic organizations in Japan about experiences of each organization regarding the adoption of cancer-related clinical practice guidelines in Japan, the academic services which each organization provides to evaluate the clinical impact and adoption of the guidelines, and the current status of recent clinical research. The questionnaire consisted of 17 questions associated with clinical practice guidelines, six questions about conflicts of interest, 15 questions about cancer registries, seven questions about clinical research/analysis, six questions related to medical information ethics, and four questions related to funding. In this study, we focused on the specific domain of the questionnaire regarding clinical practice guidelines for the treatment of cancer.

The purpose of this study was to clarify the current status of each academic organization in developing clinical practice guidelines for the treatment of cancer in Japan, and to propose recommendations for the development of clinical guidelines in the future.

## Subjects and methods

### Subjects

A search using two key terms, “cancer” and “clinical practice guidelines,” was conducted in the databases of the National Cancer Center Japan, the Japan Society of Clinical Oncology, and the Medical Information Network Distribution Service (Minds) in November 2016 to identify academic organizations which were responsible for publishing clinical practice guidelines dealing with “cancer.” We identified 37 guidelines in 28 organizations (Table 1). We conducted a survey of 28 presidents or chairpersons from the organizations which develop cancer-related clinical practice guidelines using a questionnaire.

**Table 1:** List of academic organizations in Japan which published cancer-related clinical practice guidelines

#	Academic organizations
1	The Research Group for Rare Neoplasms of Japan
2	Japanese Society for Cancer of the Colon and Rectum
3	Japanese Gastric Cancer Association
4	The Japan Society of Hepatology
5	Japanese Society of Hepato-Biliary-Pancreatic Surgery
6	Japan Society of Clinical Oncology: Proper management using Antiemetic agent
7	Japan Society of Clinical Oncology: Proper management using G-CSF
8	Japanese Society for Palliative Medicine
9	Japanese Society of Hematology
10	Japanese Society of Oral Oncology
11	Japanese Society of Thyroid Surgery
12	The Japanese Society of Pediatric Hematology Oncology
13	The Japan Esophageal Society
14	Japan Neuroendocrine Tumor Society
15	Japan Pancreas Society
16	The Japanese Orthopedic Association
17	Japan Society for Head and Neck Cancer
18	Japanese Breast Cancer Society
19	Japan Association of Breast Cancer Screening
20	The Japan Society for Neuro-Oncology
21	The Japan Lung Cancer Society
22	The Japanese Urological Association
23	Japanese Skin Cancer Society
24	Japan Society of Gynecologic Oncology
25	Japanese Society for Radiation Oncology
26	The Japanese Association of Rehabilitation Medicine
27	Japanese Society of Medical Oncology
28	The Japanese Lymphedema Society

We analyzed the status of cancer-related clinical practice guidelines in Japan and identified the current situation and problems. Based on our findings, we developed recommendations regarding the creation of cancer-related clinical practice guidelines in the future.

### Questionnaire

The following questionnaire was developed and implemented from October to November 2016. The questionnaire consisted of 19 questions regarding the following: (1) awareness of the national popularization; (2) clinical values of the guidelines; (3) miscellaneous situations and funds (Table 2). The questionnaire was distributed by a study group of the Ministry of Health, Labor, and Welfare (2015-Cancer countermeasure, General-003).

**Table 2:** Contents of questionnaire survey pertaining to the development and publication of cancer-related clinical practice guidelines

1	Have you ever surveyed the national popularization of clinical guidelines on cancers in your field?
1-a	If yes, what rate of national popularization of the guidelines did you achieve in the survey?
2	Have you ever investigated clinical values of “The recommendations for medical practice,” which you have presented in the guidelines?
2-a	If no, what do you think the obstacle was?
2-b	If yes, how many clinical guidelines have you investigated?
2-c	If you publish a paper about the clinical values of any recommendations for medical practice, please list them in terms of the published year
2-d	How did you register the cases that met the inclusion criteria of the research?
3	Did you have any ideas regarding the current situation where the governance and management of clinical practice guidelines is the responsibility of academic organizations?
3-a	Have you come up with any idea regarding current situations of establishing clinical guidelines on cancers in Japan?
3-b	What kind of organization do you recommend to manage making guidelines?
3-c	What kind of funds do you recommend to operating new organization?
3-d	What is your annual budget to operate the guidelines, including making and presenting?
3-e	Relations between the rate of surveillance investigating popularization and annual budget on clinical practice?
3-f	What is essential to overcome difficult situations while conducting medical quality assessment?
3-g	What are your thoughts on the current situation wherein the academic organization is responsible for the total management of establishing cancer-related clinical guidelines?
4	Do you publish information for patients about clinical guidelines or present that information on the internet?
4-a	What was your intention behind publishing clinical guidelines for the patients?
4-b	What were the difficulties faced during process optimization?
4-c	When do you plan to revise them?

## Results

All 28 academic organizations responsible for publishing clinical practice guidelines on treating cancers responded to our questionnaire (100% response rate).

### Awareness of the national popularization

Half of the academic organizations (*N* = 14/28) had information about the nationwide adoption of their clinical practice guidelines (Table 3: Question 1), with 79% of organizations (*N* = 11/14) reporting that the nationwide adoption rate of their guidelines was 41% or more (Table 3: Question 1-a). Among those, 73% of organizations (*N* = 8/11) believed that the adoption rate of their clinical guidelines was 71% or more.

**Table 3:** Awareness of the national popularization

Q1: Have you ever surveyed the national popularization of clinical guidelines on cancers in your field (*N* = 28)?		
Yes, we have (*N* = 14; 50%)		
No, we have not yet (*N* = 14; 50%)		

Q1-a: If yes, what rate of national popularization of the guidelines did you achieve in the survey (*N* = 14)?		
Estimated rate (%)	*N*	%
41–50	1	7
51–60	1	7
61–70	1	7
71–80	3	22
81–90	3	22
91–100	2	14
No reply	3	22

### Clinical values of the guidelines

In this survey (Table 4: Question 2), 68% of organizations (*N* = 19/28) claimed that they had already investigated the clinical values of recommendations for medical practice which were presented in their clinical guidelines. For the nine organizations that did not conduct such an investigation, the main reasons were the lack of a registry and/or analytical systems and insufficient funds (Table 4: Question 2-a).

**Table 4:** Clinical values of the guidelines

Q2: Have you ever investigated clinical values of “The recommendations for medical practice,” which you have presented in the guidelines (*N* = 28)?		
Yes, we have (*N* = 19; 68%)		
No, we have not yet (*N* = 9; 32%)		

Q2-a: If no, what do you think the obstacle was (*N* = 9)?		
Obstacles	*N*	%
Lack of registry and/or analytical systems	9	100
Short funding	9	100
Regal problem	3	33
Ethical problem	3	33
Others	2	22

### Miscellaneous situations and funds

Most organizations were unsatisfied with the current state of clinical guidelines development in Japan. 68% of organizations (*N* = 19/28) believed that a new supervisory organization was needed to support the dissemination and management of their clinical guidelines (Table 5: Question 3-a). Only 29% of organizations (*N* = 8/28) were satisfied with the current situation.

**Table 5:** Miscellaneous situations and funds

Q3-a: Have you come up with any idea regarding current situations of establishing clinical guidelines on cancers in Japan (*N* = 28)?								
Opinions			*N*			%		
We are satisfied with the current systems			8			29		
Creating a new supervisory organization is necessary			19			68		
Others			1			3		

Q3-b: What kind of organization do you recommend to manage making guidelines (*N* = 28)?								
Opinions				*N*			%	
New organization directly under control by the government				4			21	
Existing academic organizations with coordination				11			58	
A private organization				3			16	
Others				1			5	

Q3-c: What kind of funds do you recommend to operating new organization (*N* = 19)?								
Opinions				*N*			%	
Funds from the national budget				16			84	
Funds from academic organizations themselves				3			16	

Q3-d: What is your annual budget to operate the guidelines, including making and presenting (*N* = 28)?								
Annual amount				N			%	
Less than 1 million yen				8			29	
One million yen to less than 3 million yen				13			46	
Three million yen and more				4			14	
No reply				3			11	

Q3-e: Relations between the rate of surveillance investigating popularization and annual budget on clinical practice guidelines (*N* = 25)?								
Annual budget	Investigated or under investigation				No further investigation			
	*N*	%			*N*			%
Less than 1 million yen	5	62			3			38
One million yen to less than 3 million yen	8	62			5			38
Three million yen and more	4	100			0			0

When asked for more detail about the role and structure of a new supervisory organization, 58% of organizations (*N* = 11/28) recommended the existing academic organizations with coordination, 21% felt that a new organization under the direct control by the government was optimal, and 16% recommended a private organization (Table 5: Question 3-b). Furthermore, 84% of organizations (*N* = 16/19) recommended that funding comes from Japan’s national budget (Table 5: Question 3-c).

In terms of the funding needed to disseminate and manage clinical guidelines in each organization, 46% of respondents (*N* = 13/28) reported annual costs in the range of 1–3 million yen. On the other hand, 29% of organizations (*N* = 8/28) were spending less than 1 million yen (Table 5: Question 3-d).

The relationship between the rate of surveillance to investigate the adoption and the annual budget for clinical practice guidelines was shown in Table 5: Question 3-e. All academic organizations who reported annual operating costs of 3 million yen or more had already investigated the adoption of their clinical practice guidelines. On the other hand, organizations with a budget of less than 3 million yen had an investigation rate of around 60%.

## Discussion

Various cancer-related clinical practice guidelines have been developed and disseminated by academic organizations in Japan to improve the quality of oncological care. The academic organizations also play a role in educating medical personnel on recommended clinical practices. Educational systems to communicate the recommendations found in clinical practice guidelines to those in the medical field have recently become popular. However, an adequate system for updating and revising the clinical practice guidelines after launching the initial guidelines does not exist. Therefore, we need to identify the circumstances currently facing academic organizations and what needs to be done to address areas of concern.

Working as a research team of the Ministry of Health, Labor, and Welfare, we prepared a questionnaire regarding the adoption of cancer-related clinical practice guidelines in Japan, the academic services in each specific organization to evaluate the clinical impact and adoption of the guidelines, and the status of recent clinical research. Our aims were to determine the current status of the development of cancer-related clinical practice guidelines in Japan and to develop recommendations for the future.

It is necessary to investigate the national popularization of clinical practice guidelines after they are disseminated. It is also important to investigate whether medical recommendations presented in the guidelines are being followed, and to verify the outcomes in clinical practice. These processes are a plan-do-check-act cycle of continual improvement and help to establish the essential systems to revise guidelines and to continually improve the quality of oncological management of patients [5].

Based on the results of this questionnaire, organizations that conducted surveys on the nationwide adoption of their clinical guidelines found relatively high rates of adoption. However, approximately 60% of organizations had insufficient annual funding for the management of their clinical practice guidelines. Approximately 30% of organizations did not conduct any evaluation to determine the extent to which their recommended medical practices were being implemented. The establishment of a registration and analysis system and securing adequate funding were cited as possible solutions to these problems and will be challenges for the future.

Regarding the management of cancer-related clinical practice guidelines, it was confirmed that many organizations agree with the need to create a new nationwide supervisory organization. The preferred structure was to have a national government commission as a third-party supervisory organization. Most respondents felt that a representative, existing, cross-sectional academic organization would be more appropriate than an organization under the direct control of the Ministry of Health, Labor, and Welfare. Specifically, the Japan Society of Clinical Oncology, which has long led a comprehensive effort aimed at the dissemination and development of new Japanese cancer-related clinical practice guidelines, was named as a candidate. A consortium of organizations that contribute to the fields of hematological neoplasm or drug therapy, including the Japanese Society of Medical Oncology, is also a potential candidate.

However, it also may be possible to directly operate a wide-ranging supervisory body at the nationwide level, as is done in the US. In this case, structure and regulations that ensure freedom in the utilization system become essential [6]. An earlier survey [7] found that the Japan Society of Clinical Oncology was again considered the most appropriate third-party organization for this role. Going forward, we hope to proceed with investigations into an organization that will play a central role in the development of cancer-related clinical practice guidelines. Two important issues that remain after the analysis of survey responses include the following: (1) substantial differences seen among the academic organizations with respect to trends in the business situation regarding the dissemination/evaluation of cancer-related clinical practice guidelines and (2) accessibility of data for additional clinical studies to provide more reliable and validated medical evidence. To solve these problems, we are continuing our efforts as a study group, and we are aspiring to achieve concrete improvements. Sufficient funds must be available to support the project to maintain and revise the guidelines. Furthermore, legal and ethical issues should be solved before establishing any registry system.

## Conclusion

Cancer-related clinical practice guidelines in Japan have increased in popularity over time. However, investigations of the rates of adoption and implementation of the clinical recommendations in the guidelines and the impact and clinical outcomes after the publication of clinical guidelines were still lower than acceptable. The reasons for this situation were inadequate budget and a lack of systems for registry and analysis. The important issues that were revealed in this study include large gaps among academic organizations regarding adoption and the assessment of the practice guidelines. Furthermore, a new organization in Japan, funded by the government, is needed to support the management of the current cancer-related clinical practice guidelines and the development of additional guidelines.

## References

[1] Field MJ, Lohr KN (1990). Clinical practice guidelines: directions for a new program.

[2] Grimshaw JM, Russell IT (1993). Effect of clinical guidelines on medical practice: a systematic review of rigorous evaluations. Lancet.

[3] Worrall G, Chaulk P, Freake D (1997). The effects of clinical practice guidelines on patient outcomes in primary care: a systematic review. Can Med Assoc J.

[4] Shimbo T, Fukui T, Ishioka C (2010). Quality of guideline development assessed by the evaluation committee of the Japan society of clinical oncology. Int J Clin Oncol.

[5] Ansari S, Rashidian A (2012). Guidelines for guidelines: are they up to the task? A comparative assessment of clinical practice guideline development handbooks. PLoS One.

[6] Winn RJ (2000). The NCCN guidelines development process and infrastructure. Oncology.

[7] Furuhata T, Hirata K, Wakao F (2014). Questionnaire survey for the development and publication of cancer clinical practice guidelines in Japan. Int J Clin Oncol.

